# Transcriptional Regulation of Cardiac Development and Disease

**DOI:** 10.3390/ijms23062945

**Published:** 2022-03-09

**Authors:** Nicole Wagner, Kay-Dietrich Wagner

**Affiliations:** Institute of Biology Valrose, University of Nice Sophia Antipolis, 06107 Nice, France; kwagner@unice.fr

The heart, which is the first organ to develop in the embryo, is indispensable for vital functions throughout life. Cardiovascular diseases are the leading cause of mortality worldwide, indicating the importance of the proper development and function of this very specialized muscle. The complex biological events in cardiac development, including very different cell types which contribute to heart formation and the action of transcriptional regulators, are often recapitulated in the cardiac repair mechanisms upon cardiac disease. A profound understanding of cardiac development is therefore necessary to develop efficient therapeutic strategies for cardiac diseases. The present Special Issue of *International Journal of Molecular Science* analyzes transcriptional regulation processes, cardiac-tissue complexity regarding advances in cardiac tissue regeneration and novel aspects in human cardiac diseases, thereby providing new insights in features of cardiac development and diseases.

In this Special Issue, Yun Wang et al. describe that the bone morphogenetic protein (Bmp) signaling pathway directly regulates the basic helix–loop–helix (bHLH) transcription factor Hand1 in the development of the cardiac outflow tract (OFT). OFT defects are the most common congenital heart defects. OFT formation is mainly orchestrated by two different progenitor cell populations, namely the second heart field (SHF) progenitors and cardiac neural crest cells (NCCs). Using an inducible conditional Bmp2 and Bmp4 double-knockout SHF cell-type specific approach, the authors elegantly demonstrated that Bmp signaling regulates Hand1 expression. Although the knockout of Bmp signaling concerns only the SHF progenitor cells, a reduced Hand1 expression was also found in NCC progenitor cells, implying that Bmp signaling regulates Hand1 in OFT formation through cell-autonomous and non-cell-autonomous mechanisms. In contrast to Bmp loss-of-function, Bmp gain-of-function increased Hand1 expression, which further confirmed that Hand1 expression responds to the Bmp signaling dosage. Smad transcription factors are the major signal transducers for receptors of the Bmp signaling pathway and can interact with specific DNA motifs to regulate gene expression. The authors further confirmed the transcriptional activation of Hand1 by Bmp/Smad using transfection assays and by the direct binding of Bmp/Smad to Hand1 with chromatin immunoprecipitation (ChIP) experiments. This study demonstrates well that the canonical Bmp/Smad signaling pathway in the SHF directly activates Hand1 expression in a dose-dependent manner during OFT development, providing novel insights into the molecular regulation of OFT development [1].

Wilms’ tumor 1 (Wt1) gene, which encodes a zinc finger protein, is an important regulator during embryogenesis but is also involved in pathological processes, such as carcinogenesis [2]. It is important to note that Wt1 has a crucial role in heart formation, as the homozygous deletion of Wt1 in mouse embryos has been proven to be lethal due to disturbed cardiac development [3]. Furthermore, Wt1 expression in the heart has been described in various cell types, including epicardial, endothelial and smooth muscle cells, and fibroblasts. In this Special Issue, we present a review that provides an overview of general cardiac development and summarize the current knowledge regarding the expression and function of Wt1 in heart development and disease in detail. We focus on the expression of Wt1 in different cardiac cell types and its regulatory mechanisms. We further detail the implication of Wt1 in human cardiac pathologies. Given the importance of Wt1 for cardiac development, it seems obvious that Wt1 is strongly involved in cardiac repair after injury, as the re-activation of developmental programs can be considered to be a paradigm for regeneration. The understanding of the role of Wt1 in these processes and the molecules involved therein is essential for the development of therapeutic strategies [4]. As Wt1 expression in cardiomyocytes remains a controversial issue in the developing and/or diseased heart and has not been reported in adult healthy hearts, we focused our research work for this Special Issue on the potential expression and function of Wt1, specifically in cardiomyocytes. We first investigated cardiac Wt1 expression levels during development, in the adult, and under pathological conditions after myocardial infarction. We found that Wt1 expression was elevated during heart development and declined after birth. Interestingly, we were able to show that Wt1 was highly expressed in cardiomyocytes during development and persisted in some adult cardiomyocytes, probably suggesting that low levels of Wt1 expression are sufficient to maintain a cardiac progenitor subset from terminal differentiation. Following myocardial infarction, the number of Wt1-expressing cardiomyocytes, as well as individual nuclear Wt1 expression in cardiomyocytes, was strongly upregulated. We further used mouse embryonic stem cell (mESC) differentiation in vitro to obtain additional insights into the Wt1 function in the process of cardiomyocyte development. It was found that Wt1 expression levels increased along with the cardiac differentiation of mESCs. We showed that Wt1 overexpression reduces the phenotypic cardiomyocyte differentiation of ES cell clones, keeping the cells in a more progenitor-like stage which is associated with modified expression levels of stem cell and cardiomyocyte marker genes [5].

Additionally, the Hippo-Yap pathway is strongly implicated in cardiac development. For this Special Issue, Zhiquiang Lin et al. investigated the regulation of cardiac Toll-like receptor genes by the YAP/TEAD1 complex, the terminal effector of Hippo-Yap signaling. Toll-like receptors (TLRs) are involved in the pathogenesis of heart failure as they modulate innate immune responses. The authors determined that the expression of TLRs postnatally increases with age and is strongly induced in pressure overload (PO) and ischemia/reperfusion (IR) stressed mouse hearts. They demonstrate that the YAP/TEAD1 complex is a repressor of cardiac TLR genes, as TEAD1 directly bound genomic regions adjacent to Tlr1, Tlr2, Tlr3, Tlr4, Tlr5, Tlr6, Tlr7 and Tlr9. Furthermore, cardiomyocyte-specific YAP depletion in vivo increased the expression of most TLR genes. This was accompanied by an increase in pro-inflammatory cytokines and an increased susceptibility and worsened outcome in response to LPS-induced stress. In conclusion, Hippo-Yap signaling helps to impede the cardiomyocyte innate immune responses upon cardiac stress [6].

Additionally, Ashraf Yusuf Rangrez et al. sought to elucidate a potential role of the SH3 domain-binding glutamic acid-rich (SH3BGR) gene in cardiomyocyte pathophysiology. They demonstrated an upregulation of SH3BGR in human and mouse cardiac hypertrophy samples. Using in vitro overexpression and knockdown experiments for SH3BGR in neonatal rat cardiomyocytes, they observed that enhanced levels of SH3BGR favor cellular hypertrophy as well as an increase in hypertrophic markers. Knockdown of SH3BGR caused a decrease in hypertrophic marker expression and cell viability, accompanied by an activation of apoptosis. On a molecular level, the authors collected evidence supporting the idea that SH3BGR mediates these effects via the induction of Serum response factor (SRF) signaling [7].

Arrhythmogenic cardiomyopathy (ACM) is caused by mutations in genes predominantly encoding for desmosomal proteins, but Lamin A/C gene (*LMNA*) mutations are also frequently observed in patients with ACM [8]. Veronique Lachaize and coworkers detailed, for this Special Issue, the biophysical and biomechanical impact of the *LMNA* D192G missense mutation on neonatal rat ventricular fibroblasts (NRVF). They evidenced a decreased elasticity, a disturbed cytoskeleton organization and altered cell-to-cell adhesion properties in the cardiac fibroblasts with *LMNA* D192G mutation [9]. As similar observations have been made before in cardiomyocytes with a mutation of *LMNA* D192G [10], the recent findings of Lachaize et al. clearly indicate the importance of *LMNA* mutations for ACM, as in addition to cardiomyocytes, cardiac fibroblasts are also highly biomechanically impacted by this mutation [9].

Cardiac regeneration studies are widely employed and aim to repair irreversibly damaged heart tissue, and often include stem-cell therapy. Daiva Bironaite et al. investigated the ability of human dilated myocardium-derived human mesenchymal stem/stromal cells (hmMSC) and their healthy, non-dilated myocardium-derived counterparts, to differentiate into a cardiomyogenic cell type. Dilated hmMSCS expressed higher levels of Histone deacetylase (HDAC) compared to hmMSCS from non-dilated myocardium. An inhibition of HDAC resulted in the downregulation of focal adhesion kinase (PTK2), and an increased expression of the cardiomyogenic differentiation-specific genes alpha cardiac actin (ACTC1) and cardiac troponin T (TNNT2), which were more pronounced in dilated hmMSCS. These data indicate that hmMSCS from pathological cardiac material might be useful in cardiac regeneration attempts once pharmacologically re-tuned [11].

Gavin Y. Oudit’s group investigated the transcriptomic modifications occurring in human end-stage dilated human cardiomyopathy (DCM), also emphasizing the transcriptome changes that occur with left ventricular assist device support (LVAD). On a histological level, the authors observed an enhanced fibrosis and larger cardiomyocyte cross-sectional areas in DCM tissues without LVAD. On the RNA level, they observed a higher expression of hypertrophic markers brain natriuretic peptide (BNP) and β-myosin heavy chain (β-MHC) in DCM without LVAD. Gene expression analysis using microarrays evidenced oxygen homeostasis, immune response and cellular growth, proliferation, and apoptosis as the most enriched pathways in both ventricles of end-stage DCM hearts. For left ventricles, they further observed a differential expression of genes implicated in the circadian rhythm, muscle contraction, cellular hypertrophy, and extracellular matrix (ECM) remodeling, whereas in right ventricles, genes involved in apelin signaling were differentially expressed. Upon LVAD, the authors observed a normalization of the immune response genes in both ventricles, while the expression of ECM remodeling and oxygen homeostasis genes improved specifically in the left ventricle. Furthermore, the expression level of four miRNAs returned to normal. This study contributes to a completion of transcriptomic analysis in human heart disease and provides valuable information not only regarding the impact of DCM on both ventricles but especially in the context of LVAD [12].

Karine Tadevosyan and coauthors present a highly interesting and detailed review in this Special Issue, entitled “Engineering and Assessing Cardiac Tissue Complexity”. They present the general approaches used to develop functional cardiac tissue, which includes a description of cardiac-tissue engineering systems, cell sources, maturation and a functional assessment of the procedures applied. This review will help researchers who seek to conduct or improve cardiac-tissue engineering [13].

Finally, Yevgeniy Kim from Arman Saparov’s group reviews the latest findings on gene therapies in cardiovascular diseases. Recent applications, benefits and pitfalls from several preclinical and clinical studies are discussed and ongoing trials are described. The limitations of the current clinical approaches for gene therapy in cardiovascular diseases are presented and valuable ways of improvement are suggested [14].

## Conclusions

Taken together, the works included in this Special Issue “Transcriptional Regulation of Cardiac Development and Disease” comprise the most recent studies that elucidate transcriptional mechanisms in cardiac development, repair, and regeneration, as well as recent advances and perspectives in the treatment of cardiovascular diseases. The articles in this Special Issue further improve our understanding of cardiac physiology and pathology, which will assist in the continuous development of efficient cardiovascular therapies in the future.

## Data Availability

Not applicable.
